# Protein Secondary Structure Prediction Based on Data Partition and Semi-Random Subspace Method

**DOI:** 10.1038/s41598-018-28084-8

**Published:** 2018-06-29

**Authors:** Yuming Ma, Yihui Liu, Jinyong Cheng

**Affiliations:** College of Information, Qilu University of Technology(Shandong Academy of Sciences), Jinan, China

## Abstract

Protein secondary structure prediction is one of the most important and challenging problems in bioinformatics. Machine learning techniques have been applied to solve the problem and have gained substantial success in this research area. However there is still room for improvement toward the theoretical limit. In this paper, we present a novel method for protein secondary structure prediction based on a data partition and semi-random subspace method (PSRSM). Data partitioning is an important strategy for our method. First, the protein training dataset was partitioned into several subsets based on the length of the protein sequence. Then we trained base classifiers on the subspace data generated by the semi-random subspace method, and combined base classifiers by majority vote rule into ensemble classifiers on each subset. Multiple classifiers were trained on different subsets. These different classifiers were used to predict the secondary structures of different proteins according to the protein sequence length. Experiments are performed on 25PDB, CB513, CASP10, CASP11, CASP12, and T100 datasets, and the good performance of 86.38%, 84.53%, 85.51%, 85.89%, 85.55%, and 85.09% is achieved respectively. Experimental results showed that our method outperforms other state-of-the-art methods.

## Introduction

Proteins play a key role in almost all biological processes; they are the basis of life. For example, they take part in maintaining the structural integrity of the cell, transport and storage of small molecules, catalysis, regulation, signaling, and the immune system. There are 20 different amino acids that form proteins in nature^1^. The amino acids of a protein are connected in sequence with the carboxyl group of one amino acid forming a peptide bond with the amino group of the next amino acid. Protein structure is essential for the understanding of protein function. In order to recognize the protein functions of proteins at a molecular level, it is sometimes necessary to determine their 3D structure. Accurately and reliably predicting structures from protein sequences is one of the most challenging tasks in computational biology^2^. Protein secondary structure prediction provides a significant first step toward tertiary structure prediction, as well as offering information about protein activity, relationships, and functions.

Protein secondary structure refers to the local conformation proteins’ polypeptide backbone. There are two regular secondary structure states, α-helix (H) and β-strand (E), and one irregular secondary structure type, the coil region (C). Sander developed a secondary structure assignment method Dictionary of Secondary Structure of Proteins (DSSP)^3^, which automatically assigns secondary structure into eight states (H, E, B, T, S, L, G, and I) according to hydrogen-bonding patterns. These eight states are often further simplified into three states of helix, sheet and coil. The most widely used convention is that helix is designated as G, H and I; sheet as B and E; and all other states are designated as a coils. Most commonly, the secondary structure prediction problem is formulated as follows: given a protein sequence with amino acids, predict whether each amino acid is in the α-helix (H), β-strand (E), or coil region (C). Protein secondary structure prediction is usually evaluated by Q3 accuracy, which measures the percentage of residues for three-state secondary structures to determine whether they have been predicted correctly.

Protein secondary structure prediction began in 1951 when Pauling and Corey predicted helical and sheet conformations for protein polypeptide backbones, even before the first protein structure was determined^2^. Many statistical approaches and machine learning approaches have been developed to predict secondary structure. One of the first approaches for predicting protein secondary structure, uses a combination of statistical and heuristic rules^4,5^. The GOR^6^ method formalizes the secondary structure prediction problem within an information-theoretic framework. Position specific scoring matrix (PSSM)^7^ based on PSIBLAST^8^ reflects evolutionary information and has made the most significant improvements in protein secondary structure prediction. Many machine learning methods have been developed to predict protein secondary structure, and exhibit good performance by exploiting evolutionary information, as well as statistic information about amino acid subsequences^9^. For example, many neural network (NN)^10–14^ methods, hidden Markov model (HMM)^15–17^, support vector machines (SVM)^18–21^, and K-nearest neighbors^22^ have had substantial success, and Q3 accuracy has reached to 80%. The prediction accuracy has been continuously improved over the years, especially by using hybrid or ensemble methods and incorporating evolutionary information in the form of profiles extracted from alignments of multiple homologous sequences^23^. Recently, several papers used deep learning networks^24–28^ to predict protein secondary structure and obtained good success. The highest Q3 accuracy without relying on structure templates is now at 82–84%^3^. DeepCNF^27^ is a deep learning extension of conditional neural fields (CNF), which integrates conditional random fields and shallow neural networks. The overall performance of DeepCNF is significantly better than other state-of-the-art methods, breaking the long-lasting ~80% accuracy. Recently SPIDER3 improved the prediction of protein secondary structure by capturing non-local interactions using long short-term memory bidirectional recurrent neural networks^29^. In the paper^30^, a new deep inception-inside-inception network, called MUFOLD-SS, was proposed for protein secondary structure prediction. SPIDER3 and MUFOLD-SS achieved better performance, compared to DeepCNF.

**Figure 1:**
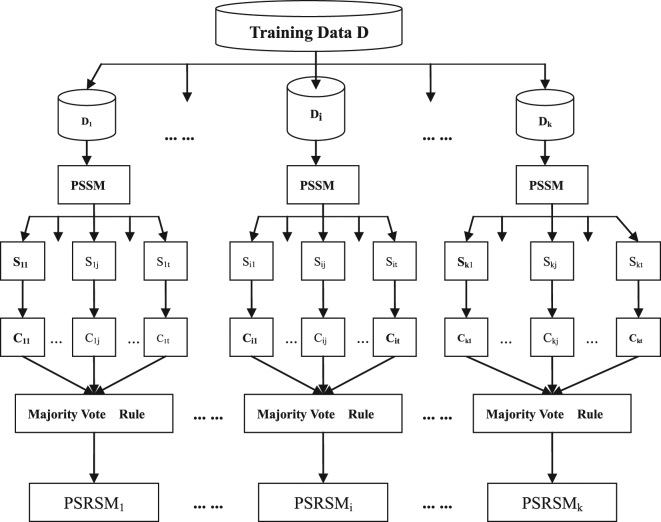
PSRSM framework. Training Data D is partitioned into k subsets D_1_, D_2_,…, D_i_, … D_k_, and S_ij_ is the jth subspace data of subset D_i_; C_ij_ is a base classifier trained on S_ij_.

In this paper, we presented a data partition and semi-random subspace method (PSRSM) for protein secondary structure prediction. The first step was partitioning the protein training dataset into several subsets based on the lengths of proteins sequences. The second step was generating subspaces by the semi-random subspaces method, training base classifiers on the subspaces, and then combining them by majority vote rule on each subset. Fig. 1 demonstrates our PSRSM experimental framework.

A key step of our method was to partition the training dataset into several subsets according to the length of the protein. The length of a protein sequence is the number of amino acids (AAs) in a protein sequence. Then we trained base classifiers in parallel on subspace data generated by using semi-random subspace method and combined them on each subset. In the conventional random subspace method, the low-dimensional subspaces are generated by random sampling of the original high-dimensional spaces. In order to get good performance of the ensemble, in this paper, we proposed a semi-random subspace method for protein secondary structure prediction. This method ensured that the base classifiers were as accurate and diverse as possible. We used support vector machines (SVMs) as the base classifier. Support vector machines are a popular machine learning method for classification, regression, and other learning tasks. Compared to other machine learning methods, SVM has the advantages of high performance, absence of local minima, and ability to deal with multidimensional datasets, in which with complex relationships exist among data elements. Support vector machines (SVMs) have had substantial success in protein secondary structure prediction.

Experimental results show that the overall performance of PSRSM was better than the current state-of-the-art methods.

## Results

### Datasets

We used six publicly available datasets (1) ASTRAL^31^, (2) CullPDB^32^, (3) CASP10^33^, (4) CASP11^34^, (5) CASP12^35^, (6) CB513^36^, and (7) 25PDB^37^ (8) a dataset T100 developed in-house. ASTRAL, ASTRAL + CullPDB, and T100 datasets are available from supplement files.

In this research, we combined the ASTRAL dataset and CullPDB dataset to be our training dataset, i.e., the ASTRAL + CullPDB dataset. The CullPDB dataset was selected based on the percentage identity cutoff of 25%, the resolution cutoff of 3 angstroms, and the R-factor cutoff of 0.25. There are 12,288 proteins in the CullPDB dataset. ASTRAL dataset had 6,892 proteins, with less than 25% sequence identity. Our training dataset ASTRAL + CullPDB had 15,696 proteins; we removed all duplicated proteins.

Publicly available datasets CASP10, CASP11, CASP12, CB513, and 25PDB were used to evaluate our method and compared using SPINE-X^38^, JPRED^39^, PSIPRED^40^ and DeepCNF. 99 proteins of the CASP10 dataset, 81 proteins of the CASP11 dataset, and 19 proteins of the CASP12 dataset were selected according to the availability of crystal structure. The CB513 dataset has 513 protein sequences. Any two proteins of CB513 share less than 25% sequence identity with each other. The 25PDB dataset was selected with low sequence similarity of no more than 25%, and has 1673 proteins, consisting of 443 all-α, 443 all-β, 346 α/β and 441 α + β. Note that the number of proteins in these datasets may be different from those reported in other published papers because we only used the available online (http://www.rcsb.org/) or with the PSSM program.

In addition, we randomly downloaded 100 new proteins (T100) released after 1 January 2018 from http://www.rcsb.org/. The dataset (T100) contains 100 proteins with sequence lengths ranging from 18 to 1460. We used T100 to test PSRSM and deepCNF using our online servers and their online server RaptorX-Property which was ranked first in secondary structure prediction.

Because T100 dataset is released after 2018, there is no duplicated proteins with our training dataset. All our training datasets were collected before February 2017.

### Performance measures

Several different measures can be used to measure the secondary structure prediction accuracy, the most common being Q3. The Q3 accuracy is defined as the percentage of residues for which the predicted secondary structures are correct, Q3 is calculated as follows:

1 $$Q3=\frac{{N}_{H}+{N}_{E}+{N}_{C}}{N}\times 100,$$

where, N_H_, N_E_, and N_C_, are the number of correctly predicted secondary structures: helix, strand and coil, respectively. *N* is the total number of residues (amino acids).

We calculate the average accuracy of the whole test dataset and use average Q3 to evaluate the performance of our model on the test dataset, the average Q3 is defined as

2 $${\rm{Average}}\,{\rm{Q}}3=\frac{{\sum }_{{\rm{i}}=1}^{{\rm{n}}}{\rm{Q}}3({{\rm{X}}}_{{\rm{i}}})}{{\rm{n}}}$$

Where n is the number of protein sequences that has the valid predicted results in the test dataset, X_i_ denotes a protein sequence, and Q3(X_i_) is the Q3 accuracy of X_i_.

### Performance

We used Q3 accuracy to compare our PSRSM method with other state-of-the-art methods, SPINE-X, PSIPRED, JPRED, and DeepCNF, on four publicly available datasets **(**CASP10, CASP11, CASP12, and CB513). Table 1 shows the Q3 accuracy of PSRSM and the other state-of-the-art methods on the four datasets. The experimental results show that PSRSM is significantly outperforming SPINE-X, PSIPRED, and JPRED. Moreover, PSRSM had 1–3% higher Q3 accuracy than DeepCNF. We also tested our method on 25PDB dataset with 1674 proteins, and Q3 accuracy is 86.38%.

**Table 1:** Q3 accuracy of the tested methods on CASP10, CASP11, CASP12, and CB513 datasets. (The results of SPINE-X, PSIPRED, JPRED, and DeepCNF are taken from the papers^2,27^).

Methods	Q3(%)			
	CASP10	CASP11	CASP12	CB513
SPINE-X	80.7	79.3	76.9	78.9
PSIPRED	81.2	80.7	78.0	79.2
JPRED	81.6	80.4	75.1	81.7
DeepCNF	84.4	84.7	82.1	82.3
PSRSM	85.51	85.89	85.55	84.53

In addition, we compared our proposed method to DeepCNF using our online servers (http://210.44.144.20:82/protein_PSRSM/default.aspx) and their online server RaptorX-Property (http://raptorx.uchicago.edu/StructurePropertyPred/predict/) on T100 dataset. Table 2 lists the Q3 accuracy of PSRSM and DeepCNF for each protein. The average Q3 accuracy of PSRSM was higher 2.5% than that of DeepCNF. In addition, we analyzed Q3 accuracy of predicted secondary structures in internal regions and at boundaries^2^. Here, we defined a helical/sheet residue as internal if its two nearest neighboring residues were also helical/sheet residues; we defined it as a boundary if one or both of the nearest neighbors had a different secondary structural assignment. The overall Q3 accuracies of PSRSM and DeepCNF, respectively, were 89.89% and 85.68% in internal regions, and 75.33% and 73.30% at boundaries. We also compared our method with other state-of-the-art methods (SPIDER3, MUFOLD, PSIPRED and JPRED) using their online server on T100 dataset in Table 3. The newly updated MUFOLD and SPIDER3 obtained 89.28% and 88.25% in internal regions, and 74.65% and 70.72% at boundaries. We can see that PSRSM was superior to current state-of-the-art methods not only in internal regions, but also at boundaries.

**Table 2:** Q3 accuracy of PSRSM and DeepCNF for each protein in the T100.

Protein name	PSRSM (Q3%)	DeepCNF (Q3%)	Length	Protein name	PSRSM (Q3%)	DeepCNF (Q3%)	Length
5K4W_A	96.88	85.67	321	5Y5Z_A	82.70	80.45	578
5MOI_A	80.27	68.61	223	6B2N_A	71.10	87.07	263
5MOJ_A	89.24	78.30	223	6BT3_C	85.00	73.64	220
5MOK_	89.24	77.58	223	6F0E_A	86.86	80.45	312
5NA1_A	76.47	79.66	408	6F1T_G	82.45	80.85	376
5O7K_A	80.21	86.46	96	6F40_A	75.75	74.79	1460
5QAN_A	91.77	78.60	243	5GZJ_A	85.24	88.30	359
5UB4_A	83.93	84.29	280	5BK1_H	93.22	80.93	236
5VSA_A	86.62	83.44	314	5GZI_B	84.68	87.74	359
6AOK_A	85.71	75.12	217	5K4Y_A	97.19	86.56	320
6FEL_A	94.07	89.83	236	5LCP_B	95.00	—	20
6F2L_A	70.07	85.53	304	5LH4_A	99.55	87.44	223
6F0Z_A	80.13	87.70	317	5MB5_A	88.18	81.82	330
6EM0_	78.83	85.20	581	5MR9_A	81.37	71.57	102
6EHH_A	94.89	85.80	176	5NXG_A	98.05	83.27	257
5QAE_A	92.18	79.84	243	5O5I_A	72.83	90.22	92
5QAK_A	92.18	79.84	243	5V6F_A	76.81	76.81	138
6AX2_A	73.91	82.61	46	5WHI_A	93.79	90.06	161
6AZ2_A	91.70	81.22	229	5WXE_A	60.71	60.71	28
6B5G_A	94.32	89.86	493	6F1D_A	94.87	88.03	117
6B7Z_A	86.54	85.09	966	5KDB_A	96.18	86.01	393
6BB5_A	94.96	84.89	139	5KDY_A	95.42	86.26	393
6BBQ_A	76.73	89.62	520	5N2O_A	88.57	92.86	70
6FD3_A	80.67	85.33	300	5NEC_A	84.48	86.64	741
6B3G_A	87.88	87.88	99	5O3U_A	91.99	83.70	724
5XXR_A	87.12	88.64	132	5O6V_A	70.36	74.19	496
5WVM_	84.68	80.16	509	5OQZ_A	77.78	—	18
5WCT_A	63.64	73.80	187	5OYD_A	89.39	85.10	396
5W30_A	79.44	79.44	180	5UG6_A	91.28	87.25	149
5MZV_B	80.81	80.81	198	5UOE_A	94.24	86.77	990
6F73_B	62.02	78.22	574	5UOZ_A	71.43	—	21
6BVC_A	83.62	81.92	177	5V23_A	78.57	86.73	98
5M3U_	91.35	83.17	416	5VDF_A	94.52	87.67	73
5BJZ_B	97.24	85.18	398	5W92_A	71.07	78.68	197
5LUH_A	90.74	79.26	270	5WAT_A	82.22	86.36	315
5MOP_	90.13	83.41	223	5WOT_A	93.43	80.30	198
5MR5_A	80.39	72.55	102	5WOZ_A	89.86	92.03	138
5NXP_A	98.45	83.33	258	5WPX_A	79.78	78.65	89
5XEE_A	76.53	77.55	98	5XBK_A	80.77	81.01	416
5YPK_A	91.32	83.88	242	5M88_A	89.71	92.65	136
5YQW_A	87.41	79.89	532	5MNV_A	89.19	87.71	407
5YWZ_A	73.55	80.17	242	5MOS_A	99.55	87.44	223
5Z0T_A	94.03	80.38	637	5MVO_A	70.45	75.95	291
6AX6_A	79.15	81.28	235	5N1D_A	90.37	84.99	353
6BGN_A	98.33	83.33	60	5N1N_A	88.95	88.67	353
6C2I_A	74.21	79.08	411	5O5C_A	82.08	84.39	519
6C8S_A	88.13	78.63	379	5OQ1_A	85.40	81.02	137
5WDD_A	93.45	91.07	168	5ORK_B	78.41	85.51	352
6AVD_A	70.00	80.00	40	5OTY_A	73.39	77.49	342
6FO0_N	88.75	87.28	480	5URT_A	71.43	—	21

(If a protein sequence has more than 4000 or less than 26 amino acids, DeepCNF online server will report errors).

**Table 3:** PSRSM, DeepCNF, SPIDER3, MUFOLD,PSIPRED and JPRED average Q3 accuracies and Q3 accuracies in the internal regions, and at boundary regions of secondary structures on the T100.

Method	Q3(average)	Q3 (internal)	Q3 (boundary)	Website
DeepCNF	82.78	85.68	73.30	http://raptorx.uchicago.edu/StructurePropertyPred/predict/
SPIDER3	82.41	88.25	70.72	http://sparks-lab.org/server/SPIDER3/
MUFOLD	84.35	89.28	74.65	http://mufold.org/mufold-ss-angle/
PSIPRED	76.33	82.84	63.06	http://bioinf.cs.ucl.ac.uk/psipred/
JPRED	74.45	81.42	60.25	http://www.compbio.dundee.ac.uk/jpred4/index.html
PSRSM	85.09	89.89	75.33	http://210.44.144.20:82/protein_PSRSM/default.aspx

The DeepCNF method is available only to proteins with a length of [26, 4000], MUFOLD is [30,700], and JPRED is [20,800].

## Discussion

### Reason for partitioning training datasets according to protein length rather than randomly

Our training data was the ASTRAL+CullPDB dataset, which had 15,696 proteins, and 3,863,231 amino acids (AAs). Since training support vector machines on such a large dataset is a very slow process, the first step of our method was partitioning the training data into several different subsets and training SVMs in parallel. If we partitioned the training data randomly, it would just reduce the computation time, but not increase the prediction accuracy^41^. The length of a protein sequence is the number of amino acids in a protein sequence. Protein length is an important feature of a protein because it influences protein structure. For example, the short sequence ‘VVDALVR’ formed ‘EEEEEE’ in six proteins: 1by5_A, 1qfg_A, 1qff_A, 1fcp_A, 1fi1_A, and 2fcp_A. Their lengths are 714, 725, 725, 705, 707, and 723 respectively. Meanwhile ‘VVDALVR’ formed ‘HHHHHH’ in one protein (3vtz_A), and its length was 269. This data can be downloaded at prodata.swmed.edu/chseq.^42^. Identical amino acid sequence has different types of secondary structures in proteins of different lengths; this is because protein length can affect both local and long-range interactions of the protein. Based on the above considerations, we partitioned training datasets according to protein length to cluster proteins in the training data.

In order to validate the effectiveness of our data partitioning strategy, we conducted another experiment. We randomly generated a subset of the ASTRAL+CullPDB dataset randomly instead of according to protein length, and similarly trained SVM base classifiers on the subset. Then we combined them into an ensemble (Classifier_C). We compared Classifier_C with our PSRSM_1_, and Table 4 shows that the performance of PSRSM_1_ is quite similar to that of Classifier_C on CB513 dataset, but significantly better on subset with protein length L ∈ [1, 100]. The main difference between the two classifiers was the training set. All training proteins of PSRSM_1_ were short proteins, they had similar protein lengths, and all lengths belonged to interval [1,100]; conversely, the lengths of Classfier_C training data were randomly distributed.

**Table 4:** Comparison of classifier_C and PSRSM_1_ on CB513.

Protein length L	Q_3_(%)		Training data			
			Classifier_C		PSRSM_1_	
	Classifier_C	PSRSM_1_	Number (protein)	Number (amino acid)	Number (protein)	Number (amino acid)
[1,100]	75.48	83.25	176	10996	2260	161952
(100,200]	78.17	76.44	255	37369	0	0
(200,300]	78.60	75.83	137	34072	0	0
(300,400]	75.94	73.82	105	35529	0	0
(400,500]	75.81	72.07	63	27818	0	0
L > 500	74.01	71.23	64	42277	0	0
all	77.16	77.57	800	188061	2260	161952

Table 5 shows the performance of T100 dataset with different lengths based on 6 PSRSMs. 6 protein subsets with different lengths achieved the best performance 79.84%, 84.58%, 87.59%, 87.51%, 83.24%, and 83.93% respectively using their corresponding PSRSM.

**Table 5:** Q3 accuracy of each ensemble classifier on different proteins with different length in T100 dataset.

	PSRSM_1_	PSRSM_2_	PSRSM_3_	PSRSM_4_	PSRSM_5_	PSRSM_6_
[1,100]	79.84	63.11	62.75	63.40	62.89	64.13
(100,200]	78.19	84.58	81.02	78.99	77.18	78.16
(200,300]	74.39	78.14	87.59	78.99	75.95	75.15
(300,400]	74.00	75.63	78.80	87.51	78.62	77.64
(400,500]	74.23	76.69	77.09	80.81	83.24	77.06
L > 500	73.59	75.87	75.64	76.30	77.12	83.93

### Training time analysis

Another advantage of our method is that the training time was short. Because our training data ASTRAL + CullPDB is a large dataset, it was very slow to train the SVM classifier. We failed to train the SVM classifier on ASTRAL + CullPDB using our server.

The computational complexity to train an SVM^43^ is

3 $$O(SVM)=O({N}_{S}^{3}+{N}_{S}^{2}N+{N}_{S}{N}_{f}N),$$

Where N_S_ is the number of support vectors, N_f_ is the feature dimension, and N is the size of the training set.

After data partitioning and sampling, the number of support vectors N_S_, feature dimension N_f_, and the size of the training set N are much smaller. Furthermore, since we trained our base classifiers in parallel, the running time was reduced.

Table 6 shows the training time on each subset of the ASTRAL + CullPDB. D_1_, D_2_, …, D_5_ and D_6_ were subsets of ASTRAL + CullPDB (Table 7). We failed to train the SVM classifier on the ASTRAL + CullPDB using our server. After data partitioning but before sampling we completed training of SVM classifiers on each subset; more time was required because D_3_ had more amino acids than other subsets. When we used PSRSM, the feature dimension was decreased, and the training time was reduced.

**Table 6:** Training time on each subset of the ASTRAL + CullPDB.

Subset	No sampling	Sampling (PSRSM)
D_1_	7 days	1.5 days
D_2_	30 days	6 days
D_3_	45 days	8 days
D_4_	40 days	7 days
D_5_	15 days	3 days
D_6_	35 days	6.5 days

**Table 7:** Subsets of training data ASTRAL + CullPDB.

Subset	Protein length L	Number of proteins	Number of amino acids
D_1_	(0, 100]	2260	161952
D_2_	(100, 200]	5256	774167
D_3_	(200, 300]	3548	877583
D_4_	(300, 400]	2382	822913
D_5_	(400, 500]	1170	519422
D_6_	(500, ∞)	1058	707309

## Conclusion and Future Work

In this paper we proposed a novel method, PSRSM, to predict protein secondary structure. The first step of our method was partitioning of the training set into several subsets based on protein length. In the second step, we generated *k* ensemble classifiers using the semi-random subspace method. If given a new query protein sequence, our method would select one, and only one, ensemble classifier from *k* ensemble classifiers according to length to predict the protein secondary structure. Experimental results showed that the overall performance of PSRSM was better than that of other current state-of-the-art methods. In particular, our method PSRSM is superior to other methods not only in internal regions, but also at boundaries.

## Methods

### Partitioning the training data

We partitioned the training data into *k* different subsets according to the protein sequence length. Let X denote a protein sequence, and L denote the length of X. We set k−1 partition points of interval $(0,\,\infty )$. Let ${{\rm{r}}}_{0}=0,\,{{\rm{r}}}_{{\rm{k}}}=\infty$, and r_1_, …, r_2_ and r_k−1_ denote partition points that satisfy ${{\rm{r}}}_{0} < {{\rm{r}}}_{1} < \cdots  < {{\rm{r}}}_{{\rm{k}}-1} < {{\rm{r}}}_{{\rm{k}}}$. These partition points partition interval (0, ∞) into k intervals without intersection. Let $R=\{(0,{r}_{1}),({r}_{1},{r}_{2}),\,\cdots ,\,({r}_{k-1},\infty )\}$.

Let D denote the training data ASTRAL + CullPDB. Subsets D_1_, D_2_, …, D_k−1_ and D_k_ are defined as follows:

4 $${D}_{i}=\{X|X\in D\wedge L\in ({r}_{i-1},\,{r}_{i}\},\,i=1,\cdots ,k;$$

and, D_1_, D_2_, …, D_k−1_ and D_k_ satisfy ${\cup }_{{\rm{i}}={\rm{1}}}^{{\rm{k}}}{{\rm{D}}}_{{\rm{i}}}={\rm{D}}$, and ${{\rm{D}}}_{{\rm{i}}}\cap {{\rm{D}}}_{{\rm{j}}}=\rlap{/}{0},\,{\rm{i}}\ne {\rm{j}}{\rm{.}}$

In our experiment, we set k = 6 and

$$R=\{(0,100],(100,200],(200,300],(300,400],\,(400,500],(\mathrm{500},\infty )\}$$

Table 7 shows the number of proteins and amino acids in ${\{{D}_{i}\}}_{i=1}^{6}$.

### Training classifiers

We generated *t* random subspaces of r-dimension, and trained *t* SVM base classifiers on each subset *D*_*i*_ *t* feature subsets are used to train *t* base classifiers, and each subset had *r* features sampled from the 260-dimensional dataset.

Therefore we got k × t SVM base classifiers on *k* subsets, we denote these classifiers as a k × t matrix, where k is the number of subsets of the training data.

5 $$(\begin{array}{cccc}{C}_{11} & {C}_{12} & \cdots  & {C}_{1t}\\ {C}_{21} & {C}_{22} & \cdots  & {C}_{2t}\\ \vdots  & \vdots  & \vdots  & \vdots \\ {C}_{k1} & {C}_{k2} & \cdots  & {C}_{kt}\end{array}),$$

where C_ij_ is the SVM base classifier trained on the *j*th subspace data of subset D_i_.

We combined classifiers ${\{{C}_{ij}\}}_{j=1}^{t}$ into a final ensemble classifier by majority vote rule, and thus got *k* ensemble classifiers as the final decision on each subset. They are denoted as below.

6 $$PSRSM=(\begin{array}{cccc}Voting({C}_{11} & {C}_{12} & \cdots  & {C}_{1t})\\ Voting({C}_{21} & {C}_{22} & \cdots  & {C}_{2t})\\  & \vdots  &  & \\ Voting({C}_{k1} & {C}_{k2} & \cdots  & {C}_{kt}\end{array})=(\begin{array}{c}PSRS{M}_{1}\\ PSRS{M}_{2}\\ \vdots \\ PSRS{M}_{k}\end{array})$$

Here ‘Voting’ means combining classifiers by majority vote rule, *PSRSM*_*i*_ represents the final ensemble classifier on subset D_i_, and,

7 $$PSRS{M}_{i}=Voting((\begin{array}{cc}\begin{array}{cc}{C}_{i1} & {C}_{i2}\end{array} & \begin{array}{cc}\cdots  & {C}_{it}\end{array}\end{array}).$$

In this study The parameters *t* is set to 12 base classifiers, and the dimension of subspaces *r* is 160 in our experiment.

The publicly available LIBSVM^44^ software was used to train SVM classifiers. There are several kernel functions, commonly used in SVM: “liner”, “polynomial”, and “radial basis”. In this paper, we used the radial basis function (RBF) as kernel, the form is ${\rm{k}}({\rm{x}},{{\rm{x}}}_{{\rm{i}}})=\exp (-{\rm{\gamma }}{\Vert {\rm{x}}-{{\rm{x}}}_{{\rm{i}}}\Vert }^{2})$, where γ is a parameter. C is another parameter for SVM training; it is the regularization factor that controls the balance between low error and large divided margin. Parameters C and γ were decided using the grid search method. The optimal values of the two parameters are 0.9956 and 0.065, respectively.

### Prediction

Given a new query protein sequence X, and protein sequence length L, our method selected one and only one ensemble classifier from *k* ensemble classifiers ({PSRSM_1_, PSRSM_2_, …, PSRSM_k_}) according to the length L to predict the protein secondary structure of X. Let $\tilde{{\rm{Y}}}$ denote the prediction output by PSRSM. Then

8 $$\tilde{Y}=PSRS{M}_{i}(X)\,if\,L\in ({r}_{i-1},{r}_{i}]\,i=1,2,\cdots ,k,$$

where, *PSRSM*_*i*_ is defined as (7).

For example, if a new query protein sequence X is a short protein and ${\rm{L}}\in (0,{{\rm{r}}}_{1}]$, then the corresponding PSRSM_1_ trained on the short protein subset is used to predict its secondary structure. In general, if ${\rm{L}}\in ({{\rm{r}}}_{{\rm{i}}-1},{{\rm{r}}}_{{\rm{i}}}]$, the *i*th classifier PSRSM_i_ will be selected from *k* ensemble classifiers to predict the protein secondary structure of X.

### Semi-Random Subspace Method (SRSM)

The random subspace method (RSM) is an ensemble construction technique. It was proposed by Ho in 1998^45^. RSM randomly samples a set of low-dimensionality subspaces from the whole original high-dimensional features space, then constructs a classifier on each smaller subspace and finally applies a combination rule for the final decision.

We proposed a semi-random subspace method for protein secondary structure prediction. In our research, each protein sequence was represented by a 260 × L matrix. The *i*th column vector represents features of the *i*th amino acid residue. We generated *t* feature subsets to train *t* base classifiers. Each subset had *r* features sampled from the 260-dimensional dataset.

Because the original PSSM of the associated residue is an important feature for the base classifier, those 20 dimensions in a central location of 260-dimensional data are fixed for each sampling.

Let S represent the 260-dimensional features vector, and ${\rm{S}}=({v}_{1},{v}_{2},\cdots ,{v}_{260})$. We generated t subspaces (${\{{{\rm{S}}}_{{\rm{i}}}\}}_{{\rm{i}}}^{{\rm{t}}}$) from S.*S*_*i*_ represents a feature subset sampled from S, and ${S}_{i}=({x}_{i1},\,{x}_{i2},\cdots ,\,{x}_{id},{v}_{121},{v}_{122},\cdots ,{v}_{140},{y}_{i1},{y}_{i2},\cdots ,{y}_{id})$, where (*v*_121_, *v*_122_, …, *v*_140_) are fixed in each *S*_*i*_;(*x*_*i*1_, *x*_*i*2_, …, *x*_*id*_) and (*y*_*i*1_, *y*_*i*2_, …, *y*_*id*_) are sampled from (*v*_1_, *v*_2_, …, *v*_120_) and (*v*_141_, *v*_122_, …, *v*_260_), respectively; here, r and d satisfy $r=2\times d+20$. Let *L*_*i*_ = (*x*_*i*1_, *x*_*i*2_, …, *x*_*id*_), S_0_ = (*v*_121_, *x*_122_, …, *v*_140_), and *R*_*i*_ = (*y*_*i*1_, *y*_*i*2_, …, *y*_*id*_), then ${S}_{i}={{\rm{L}}}_{{\rm{i}}}\cup {{\rm{S}}}_{0}\,\cup {{\rm{R}}}_{{\rm{i}}}$.

Additionally, high diversity of base classifiers can make an ensemble with more accurate decisions; this is because different base classifiers make different errors on different patterns. The diversity of base classifiers is negatively correlated with the similarity of the training data. $|{{\rm{S}}}_{{\rm{i}}}\cup {{\rm{S}}}_{{\rm{j}}}|$ reflects the similarity of S_i_ and S_j_ to a certain extent. When $|{{\rm{S}}}_{{\rm{i}}}\cap {{\rm{S}}}_{{\rm{j}}}|$ is smaller, the similarity between S_i_ and S_j_ is smaller, and the diversity of base classifiers is higher. Let o_j_ be the number of occurrences of v_j_ in ${\{{S}_{i}\}}_{i=1}^{t}$. In our research, it can be proved that, when ${{\rm{o}}}_{{\rm{i}}}=\frac{{td}}{120}\,{\rm{for}}\,{\rm{i}}=1,2,\ldots ,120\,{\rm{and}}\,{\rm{i}}=141,142,\ldots ,260$, the sum ${\sum }_{{\rm{i}}=1}^{{\rm{t}}}{\sum }_{{\rm{j}}=1}^{{\rm{t}}}|{{\rm{S}}}_{{\rm{i}}}\cap {{\rm{S}}}_{{\rm{j}}}|$ becomes the minimum. Therefore our method was to generate *t* feature subsets randomly, and adjust elements of each subspace to make o_i_ = $\frac{{\rm{td}}}{120}$ for i = 1, 2, …, 120 and i = 141, 142, …, 260.

The steps of the proposed semi-random subspace method are as follows.

1. Generating semi- random subspaces

1. Let ${\rm{L}}=({v}_{1},{v}_{2},\cdots ,{v}_{120})$, ${{\rm{S}}}_{0}=({v}_{121},{v}_{122},\cdots ,{v}_{140})$, and ${\rm{R}}=({v}_{141},{v}_{142},\cdots ,{v}_{260})$ and generate d-dimensional random subspaces ${\{{{\rm{L}}}_{{\rm{i}}}\}}_{{\rm{i}}=1}^{{\rm{t}}}$ from L, ${\{{{\rm{R}}}_{{\rm{i}}}\}}_{{\rm{i}}=1}^{{\rm{t}}}$ from R, respectively.
2. Calculate ${\{{{\rm{o}}}_{{\rm{j}}}\}}_{{\rm{j}}=1}^{120}$, where o_j_ is the number of occurrences of v_j_ in ${\{{{\rm{L}}}_{{\rm{i}}}\}}_{{\rm{i}}}^{{\rm{t}}}$. Let ${\rm{mino}}=\,{\rm{\min }}\,{\{{{\rm{o}}}_{{\rm{j}}}\}}_{{\rm{j}}=1}^{120}$ and ${\rm{maxo}}=\,{\rm{\max }}\,{\{{{\rm{o}}}_{{\rm{j}}}\}}_{{\rm{j}}=1}^{120}$.
3. Let $${\rm{idmin}}=\{{\rm{j}}|{{\rm{o}}}_{{\rm{j}}}={\rm{mino}}\wedge {\rm{j}}=1,2,\cdots ,120\}$$ and
  ${\rm{idmax}}=\{{\rm{j}}|{{\rm{o}}}_{{\rm{j}}}={\rm{maxo}}\wedge {\rm{j}}=1,2,\cdots ,120\}$. Then generate a ternary ordered pairs set ${\rm{P}}=\{({\rm{i}},{\rm{j}},{\rm{k}})|{{\rm{v}}}_{{\rm{j}}}\notin {{\rm{L}}}_{{\rm{i}}}\wedge {{\rm{v}}}_{{\rm{k}}}\in {{\rm{L}}}_{{\rm{i}}}\wedge {\rm{j}}\in {\rm{idmin}}\wedge {\rm{k}}\in {\rm{idmax}}\}$.
4. Randomly select a ternary ordered pair (i, j, k) from P, insert feature v_j_ to L_i_, and delete v_k_ from L_i_. Then return to step (1) until ${{\rm{o}}}_{1}={{\rm{o}}}_{2}=\cdots ={{\rm{o}}}_{120}$.
5. Repeat (2), (3) and (4) on ${\{{{\rm{R}}}_{{\rm{i}}}\}}_{{\rm{i}}=1}^{{\rm{t}}}$ and R.
6. $${{\rm{S}}}_{{\rm{i}}}={{\rm{L}}}_{{\rm{i}}}\cup {{\rm{S}}}_{0}\cup {{\rm{R}}}_{i},i=1,2,\cdots ,{\rm{t}}$$.

2. Construct t classifier ${\{{{\rm{C}}}_{{\rm{i}}}\}}_{{\rm{i}}}^{{\rm{t}}}$ from the corresponding t random subspaces.
3. Combine classifiers ${\{{{\rm{C}}}_{{\rm{i}}}\}}_{{\rm{i}}}^{{\rm{t}}}$ by majority vote rule.

There are two parameters to be determined for the semi-random subspace method, i.e., the number of subspaces *t*, and dimension of subspaces *r*.

**Figure 2:**
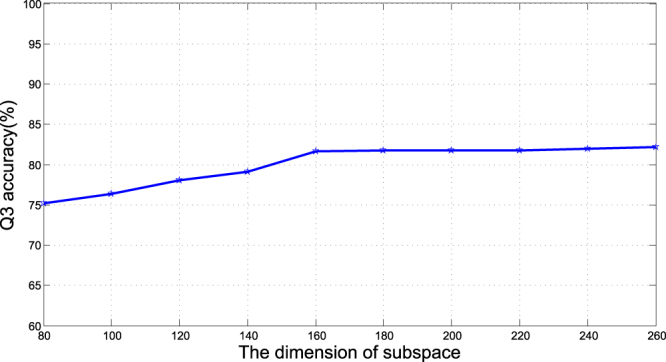
Relationship between Q3 accuracy and dimension of subspace.

Since D_1_ was smaller than other subsets, the training time on D_1_ was shorter than on other subsets. Therefore we conducted a series of experiments on D_1_ to determine *t* and *r*. We fixed t = 12, because it requires t*d to be divided by 120, it is easy to set d or *r*. Experimental results on the CB513 dataset showed that with increasing *r* the Q3 accuracy increased, but when *r* > 160, the Q3 accuracy increased slowly(Fig. 2) and the training time must be much longer. So we determine *r* = 160 as the dimension of subspaces in our experiment.

### Input features

The PSSM of a protein sequence represents homolog information affiliated with its aligned sequences. We used the PSI-BLAST program to generate the PSSM data. PSI-BLAST used BLOSUM62 evolutionary matrix to search a reduced version of the NCBI’s non-redundant (NR) database filtered at 90% sequence similarity, in order to find the variability of the residue within a multiple sequence alignment. PSI-BLAST parameters was set with threshold *h* = 0.001 and *j* = 3 iterations. The resulting PSSMs were a 20 × L matrix, where L is the protein length and 20 is the number of amino acid types.

A sliding window of consecutive amino acids was used to obtain residue sequence information and predict the secondary structure of the central residue. Each residue was encoded by a vector of dimension 20 × w, where w is the sliding window size and is an odd number. The window was shifted from residue to residue through the protein chain. In this paper, the sliding window length w was set to 13. To use the first and last six amino acids, we inserted six zeros before and behind each protein sequence. Therefore each protein sequence was represented by a 260 × L matrix, and the ith column vector represented the protein features associated with the ith residue.

Secondary structure assignment was done with the DSSP. DSSP program defines eight states for secondary structure (H, E, B, T, S, L, G, and I) that are reduced to three states (H, E, and C) by different predictive methods. We used the following reductions: H, G and I to helix (H); E and B to beta strands (E); all the rest to coil (C).

### Availability

http://210.44.144.20:82/protein_PSRSM/default.aspx.

## Electronic supplementary material

Dataset 1

Dataset 2

Dataset 3
